# Safety and Efficacy of Nonanesthesiologist-Administrated Propofol during Endoscopic Submucosal Dissection of Gastric Epithelial Tumors

**DOI:** 10.1155/2019/5937426

**Published:** 2019-01-10

**Authors:** Keiichiro Abe, Keiichi Tominaga, Akira Kanamori, Tsunehiro Suzuki, Hitoshi Kino, Masakazu Nakano, Takeshi Sugaya, Kouhei Tsuchida, Yuichi Majima, Toshimitsu Murohisa, Makoto Iijima, Kenichi Goda, Atsushi Irisawa

**Affiliations:** Department of Gastroenterology, Dokkyo Medical University, Tochigi, Japan

## Abstract

**Objective:**

There is no consensus regarding administration of propofol for performing endoscopic submucosal dissection (ESD) in patients with comorbidities. The aim of this study was to evaluate the safety and efficacy of propofol-induced sedation administered by nonanesthesiologists during ESD of gastric cancer in patients with comorbidities classified according to the American Society of Anesthesiologists (ASA) physical status.

**Methods:**

Five hundred and twenty-two patients who underwent ESD for gastric epithelial tumors under sedation by nonanesthesiologist-administrated propofol between April 2011 and October 2017 at Dokkyo Medical University Hospital were enrolled in this study. The patients were divided into 3 groups according to the ASA physical status classification. Hypotension, desaturation, and bradycardia were evaluated as the adverse events associated with propofol. The safety of sedation by nonanesthesiologist-administrated propofol was measured as the primary outcome.

**Results:**

The patients were classified according to the ASA physical status classification: 182 with no comorbidity (ASA 1), 273 with mild comorbidity (ASA 2), and 67 with severe comorbidity (ASA 3). The median age of the patients with ASA physical status of 2/3 was higher than the median age of those with ASA physical status of 1. There was no significant difference in tumor characteristics, total amount of propofol used, or ESD procedure time, among the 3 groups. Adverse events related to propofol in the 522 patients were as follows: hypotension (systolic blood pressure < 90 mmHg) in 113 patients (21.6%), respiratory depression (SpO_2_ < 90%) in 265 patients (50.8%), and bradycardia (pulse rate < 50 bpm) in 39 patients (7.47%). There was no significant difference in the incidences of adverse events among the 3 groups during induction, maintenance, or recovery. No severe adverse event was reported. ASA 3 patients had a significantly longer mean length of hospital stay (8 days for ASA 1, 9 days for ASA 2, and 9 days for ASA 3, *P* = 0.003). However, the difference did not appear to be clinically significant.

**Conclusions:**

Sedation by nonanesthesiologist-administrated propofol during ESD is safe and effective, even for at-risk patients according to the ASA physical status classification.

## 1. Introduction

Endoscopic submucosal dissection (ESD) is a minimally invasive treatment that has a high en bloc resection rate and has been widely accepted as a standard treatment for early gastrointestinal cancer [1–4]. Without adequate sedation, ESD procedure may cause unnecessary suffering to patients and even lead to serious adverse events such as perforation due to unexpected body movements. Therefore, maintaining the intended level of sedation is essential to performing ESD safely. Traditionally, benzodiazepines have been widely used for sedation during ESD. More recently, the usefulness of propofol has been increasingly reported [5, 6]. Propofol can be administered continuously because it has a much shorter half-life compared with benzodiazepines and exhibits almost no accumulation effect. For maintaining sedation over a long period of time, this is a major advantage [7]. The safety of propofol in elderly patients has already been reported [8]. Careful attention is required when using propofol in patients with multiple comorbidities because its use is known to be associated with risks of respiratory and circulatory depression.

The American Society of Anesthesiologists (ASA) physical status classification was developed as a means of defining preoperative risk. Correlation between ASA classification and perioperative mortality has been reported [9]. A previous report showed that sedation using benzodiazepines for ESD is safe and effective in patients with any ASA physical status classification [10]. In contrast, there has been no reported study on the safety and efficacy of propofol during ESD in patients with comorbidities according to ASA physical status classification, especially when administration by nonanesthesiologists (endoscopists). In this study, we retrospectively evaluated the safety and efficacy of propofol-induced sedation administered by nonanesthesiologists during ESD for gastric cancer in patients with comorbidities classified according to their ASA physical status.

## 2. Patients and Methods

### 2.1. Study Design

This study was designed as a retrospective evaluation based on the medical records of Dokkyo Medical University Hospital. The institutional review board approved this study. Because the patient's data was retrospectively analyzed in this study, we provided a means to “opt out” instead of written and signed informed consent, which is a way to guarantee the opportunity for research subjects to notify and to withdraw their medical records from data analysis if they wish. This study was registered with the University Hospital Medical Information Network (UMIN) registration number UMIN000033877.

The primary outcome was measured as the safety of sedation using nonanesthesiologist-administrated propofol in patients with comorbidities. The secondary outcomes were as follows: technical/clinical success of ESD, adverse event associated with the ESD procedure, and length of hospital stay.

Adverse events were divided into three groups according to their onset time: induction phase, maintenance phase, and recovery phase. The induction phase was defined as the start of propofol administration to insertion of the endoscope. The maintenance phase was defined as insertion of the endoscope to completion of dissection. The recovery phase was defined as the completion of dissection to discharge from the endoscopy unit.

### 2.2. Patients

Five hundred and twenty-two patients (564 lesions) who received ESD for gastric tumor (cancer/adenoma) under sedation via nonanesthesiologist-administrated propofol between April 2011 and October 2017, at Dokkyo Medical University Hospital, were enrolled in this study. We obtained the written and signed informed consent before the ESD procedure and administration of propofol. Indication for ESD was assessed according to the guidelines of the Japanese Gastric Cancer Association, including the extended criteria. The patients were divided into 3 groups according to the ASA physical status classification. ASA class 1 defines healthy patients (no organic, physiological, psychiatric, or biochemical disturbances). ASA class 2 includes patients with mild systemic diseases and no functional limitations (such as cerebral vessel disease, respiratory disease, ischemic heart disease, arrhythmia, chronic liver disease, chronic renal dysfunction, hypertension, or diabetes mellitus). ASA class 3 includes patients with severe systemic diseases and definite functional limitations (such as diabetes with associated vascular complications, poorly controlled hypertension, a history of myocardial infarction, or emphysema). There were no patients categorized as ASA class 4 or 5 because treatment under sedation by nonanesthesiologists in endoscopy is contraindicated for high-risk patients [11].

For patients taking antiplatelet agents who have a low risk of thromboembolism, ESD was performed 3–5 days after discontinuation of antiplatelet therapy. For patients with a high risk of thromboembolism, ESD was performed without discontinuation of antiplatelet therapy. For patients taking anticoagulants in whom anticoagulant therapy could not be discontinued due to the risk of thromboembolism, heparinization (10,000–15,000 U/body/day) was performed, and anticoagulant therapy discontinued for 6 hours before and after ESD.

### 2.3. Sedation Protocols

Before sedation, 8% lidocaine spray was used for anesthesia of pharynges. Administration of propofol was performed by an endoscopist who was not performing the ESD. The depth of sedation was adjusted to “deep sedation” under which spontaneous breathing is maintained, without using bispectral index monitoring, in accordance with the “Practice Guidelines for Sedation and Analgesia by Non-anesthesiologists” published by the American Society of Anesthesiologists (ASA). “Deep sedation” was equivalent to a score of 5 or 6 on the Ramsay sedation scale.

Sedation was induced using an infusion pump at a rate of 0.8 mg/kg/10 seconds in patients with age < 70 years and at a rate of 0.5 mg/kg/10 seconds in those with age > 70 years [12]. Additional propofol (0.5 mg/kg) was administrated as necessary. To maintain a Ramsay score of 5/6, the rate was preserved at 3 mg/kg/h. Furthermore, 15 mg pentazocine was initially administered, and 7.5 mg pentazocine was additionally given every 30 min during ESD. In the event of body movement, a bolus IV infusion of 10 mg propofol was given and the continuous infusion rate increased. If an adverse event occurred, the continuous infusion rate was decreased. After the completion of ESD, the continuous propofol infusion was stopped to allow the patient to recover from anesthesia.

Propofol was administrated by the nonanesthesiologist endoscopist who did not directly relate with the ESD procedure in all cases. Therefore, the nonanesthesiologist endoscopist participated in a training session for sedation and advanced cardiovascular life support. In addition, an anesthesiologist was on standby in case of emergency.

### 2.4. Monitoring Protocols

During ESD, patients received oxygen via a nasal cannula at a rate of 2 L/min. Our monitoring protocol was planned in accordance with the American Society for Gastrointestinal Endoscopy training guidelines for the use of propofol in gastrointestinal endoscopy [13]. The blood pressure (BP) was measured immediately after sedation, then every minute until the insertion of the endoscope, and then every 5 minutes thereafter. The heart rate (HR), electric cardiogram, and SpO_2_ were monitored continuously. A nurse and another endoscopist who were not performing the ESD checked the vital signs of the patients and recorded the data. If the SpO_2_ was below 90% for at least 10 seconds in room air, the mandible was lifted and oxygen was administered until the SpO_2_ was 95% or higher. If SpO_2_ did not improve within 3 minutes, the ESD procedure and sedation were interrupted to secure the airway. In addition, we prepared a course of bag valve ventilation for emergency. If systolic blood pressure was below 90 mmHg, the intravenous infusion rate was immediately increased (100–150 mL/h), and the propofol dose was decreased by 1 mg/kg/h. Moreover, intravenous injection of atropine (1.0 mg) was prepared for bradycardia by sedation (HR less than 45/min continued for at least 1 minute).

### 2.5. ESD Procedure

During ESD procedure, an overtube of an endoscope which was with deaeration preventive valve, inner diameter of 20 mm, and length of 210 mm (TOP Corp., Tokyo, Japan) was placed into the esophagus via the mouth for prevention of aspiration pneumonia. In addition, an intermittent leg compression device was used for the prevention of deep vein thrombosis.

ESD was performed using a dual knife (KD-650L; Olympus Corp., Tokyo, Japan) for marking and precut; IT knife (KD-610L; Olympus Corp.) or IT knife 2 (KD-611L; Olympus Medical Systems Corp.) was used for marginal cut and submucosal dissection. For submucosal injection, a 1 : 1 solution of 0.4% sodium hyaluronate (MucoUp; Johnson & Johnson K. K., Tokyo, Japan) and glycerol (Chugai Pharmaceutical Co. Ltd., Tokyo, Japan) was injected into the submucosa using a 25 G injection needle. If unexpected bleeding occurred, hemostatic forceps were used. All ESD procedures were performed by expert physicians who were board-certified gastroenterological endoscopists of the Japan Gastroenterological Endoscopy Society or by less-experienced physicians under supervision of the expert physicians.

### 2.6. Adverse Event

In this study, the following adverse events related to propofol were evaluated: hypotension (systolic blood pressure < 90 mmHg), desaturation (SpO_2_ < 90% on room air), and bradycardia (heart rate < 50 beats/min), based on the Grade 3/4 toxicity criteria [14]. Evaluated adverse events associated with the ESD procedure were as follows: postoperative bleeding and perforation. Postoperative bleeding was confirmed when hemostatic treatment by endoscopic management or blood transfusion was required within 2 weeks of the ESD procedure. Perforation was confirmed when the omentum or abdominal cavity was visible during endoscopic treatment or when free air was recognized on radiography after the treatment [15].

### 2.7. Statistical Analyses

Data in this study were expressed as means, standard deviations, or medians, as appropriate. The results were statistically analyzed using the Kruskal-Wallis test. Statistical significance was set at *P* < 0.05. Data analysis was performed using IBM SPSS Statistics 21® (IBM Japan Ltd.).

## 3. Results

### 3.1. Baseline Characteristics of the Patients

The baseline characteristics of the patients are shown in Table 1. Patients were classified into three groups: ASA 1 (*n* = 182), ASA 2 (*n* = 273), and ASA 3 (*n* = 67). The median age was 63.5 years (38–89 years) in the ASA 1 group, 72.0 years (51–93 years) in the ASA 2 group, and 72.0 years (49–95 years) in the ASA 3 group. The most common comorbidities in the ASA 2/3 groups were hypertension, heart disease, and diabetes mellitus. There were differences in age, anticoagulant/antiplatelet administration, and heparinization between the ASA 1 and ASA 2/3 groups. The percentage of patients on anticoagulant/antiplatelet agents also increased with a higher grade of ASA (4.94% for the SAS 1 group, 27.4% for the ASA 2 group, and 58.2% for the ASA 3 group; *P* < 0.001). There were no differences in the site of the target lesion for ESD among the three groups (*P* = 0.141).

### 3.2. Detail of Propofol Administration

Details of propofol administration are shown in Table 2. There was no difference in procedure time among the three groups (122 ± 68.9 min for the ASA 1 group, 114 ± 66.8 min for the ASA 2 group, and 109 ± 45.1 min for the ASA 3 group; *P* = 0.216). There were also no differences in total infusion dose (mg), induction dose (mg/kg/h), maintenance dose (mg/kg/h), or waking time (min) among the three groups.

### 3.3. Safety

Adverse events related to propofol in the 522 patients were as follows: hypotension (systolic blood pressure < 90 mmHg) in 113 patients (21.6%), respiratory depression (SpO2 < 90%) in 265 patients (50.8%), and bradycardia (pulse rate < 50 bpm) in 39 patients (7.47%) (Table 3). Evaluation of the frequency and types of adverse events in each treatment phase (induction, maintenance, and recovery) showed no differences in the three groups. Hypotension and bradycardia improved soon after adjusting propofol dose. Therefore, there was no patient who was administrated atropine for bradycardia. Respiratory depression tended to occur in the induction phase, but oxygen saturation improved after oxygen administration. Therefore, there was no patient who had bag valve ventilation performed. No patients developed a resedated condition after completion of propofol administration; thus, we did not need support by an anesthesiologist in all cases.

### 3.4. Adverse Events Related to the ESD Procedure and Clinicopathological Tumor Characteristics

Adverse events related to ESD procedure in the 522 patients are shown in Table 4. They were bleeding, perforation, and aspiration pneumonia. The frequency of occurrence of the adverse events was not different among the three groups.

Regarding the clinicopathological tumor characteristics, there were no differences in tumor diameter, resected specimen diameter, or en bloc resection rate among the three groups.

### 3.5. Length of Hospital Stay

The median length of hospital stay in the 552 patients was 9 days (6–35 days). Comparison among the three groups showed that the length of hospital stay tended to be longer in patients of the ASA 2 and 3 groups (8 days (6–25 days) for the ASA 1 group, 9 days (6–21 days) for the ASA 2 group, and 9 days (7–35 days) for the ASA 3 group; *P* = 0.003).

## 4. Discussion

As the population ages, endoscopists are increasingly faced with challenges associated with treating older patients with comorbidities safely and effectively [16, 17]. Currently, ESD is widespread as a minimally invasive endoscopic treatment for early gastrointestinal cancer and is also actively applied to large lesions and lesions with an ulcer scar that cannot be resected using conventional endoscopic mucosal resection (EMR) [18–20]. ESD requires precise and complicated maneuvers and, thus, results in longer operating times and increases the risks of undesirable side effects compared with EMR. Safe and stable sedation is essential in the procedure. Several studies have reported on the safety and efficacy of ESD in elderly patients [8, 21–23]. However, no studies seem to be available on the safety of sedation with propofol in gastric ESD patients with comorbidities as defined by the ASA physical status classification. In this study, we showed that sedation with propofol was safe in patients with various comorbidities and those on various medications, if appropriately administered. It is of note that, in this study, sedation was achieved by endoscopists and not by anesthesiologists. Under safe and stable sedation, ESD was successfully performed and the treatment results were favorable, irrespective of ASA classification.

In the United States, gastrointestinal endoscopists have provided sedation for endoscopic procedures without anesthesiologists since the year 2000. The safety of propofol sedation by endoscopists in endoscopic units has been reported [24–26]. Currently, it is widely accepted as an effective and safe sedation method. A previous study showed no significant difference in respiratory and circulatory depression between propofol and midazolam (a benzodiazepine) [27]. Another study demonstrated that propofol was not associated with increased risks of complications [28]. In addition, propofol has advantages, such as faster waking time due to its shorter half-life and lower risk of resedation due to the absence of accumulation effect [29].

In recent years, propofol has been increasingly used instead of midazolam. Careful attention is required when using propofol because it is an intravenous anesthetic. Being an intravenous anesthetic agent, propofol is associated with greater risks of serious adverse events such as respiratory and circulatory depression, compared with midazolam.

In our study, there were no differences in the frequency and types of propofol-related adverse events among the three ASA groups. A previous study reported that respiratory depression occurred in approximately 20% of patients, and the incidence was particularly high during the induction phase [5]. In our study, respiratory depression (oxygen desaturation) was observed during the induction phase in more than half of the cases and the incidence was higher than that in the previous study. However, all the patients improved after oxygen administration and had no further problems. This high incidence of desaturation during the induction phase might be due to delayed onset of the oxygen supply, which was not started until oxygen saturation was <90%. It was therefore assumed that desaturation may have been prevented in many cases, by providing oxygen at the same time as the start of propofol administration. Hypotension and bradycardia were observed with a certain frequency, but the symptoms improved after adjusting propofol doses. Therefore, safe and effective sedation can be achieved by endoscopists even in patients with higher ASA grade through appropriate monitoring and being responsive to adverse events.

According to the ASA physical status classification, patients classified as class 3 or higher represent high-risk [30]. In this study, there were no differences in procedure time, propofol dose, or waking time among the three groups. The length of hospital stay tended to be longer with higher ASA grades. A study of the relationship between sedatives and length of hospital stay reported that the length of stay was longer with higher ASA grade under sedation with midazolam, flunitrazepam, haloperidol, and butorphanol [10], which is similar to our result. However, in our study, the length of stay was not related to the safety and effectiveness of propofol but to the heparinization in patients receiving antithrombotic therapy. Heparinization was more frequent in patients with higher ASA grades.

Regarding ESD-related adverse events, there were no differences among the three groups. This supports the safety of the procedure. Propofol sedation in combination with intravenous opioids was reported to increase risks of aspiration pneumonia in elderly patients due to decreased pharyngeal reflexes [31, 32]. However, in this study, aspiration pneumonia did not occur even in the ASA 3 group who had moderate to severe diseases and restrictions in their daily activities. There was no association between ESD-related adverse events and types of comorbidities.

Our study has several limitations. First, this is a single-center study with a retrospective design. Second, pentazocine was used in combination with propofol in some patients, but the influence of this was not investigated. Since there was no difference in the occurrence of procedural accidents between patients who used pentazocine and those who did not, the influence of pentazocine was considered to be minimal. However, further investigation of drug interactions with combined sedatives including opioids is needed. In addition, no studies have investigated the effects of concurrent use of pentazocine on sedation. Third, we did not use the capnography for monitoring during sedation. Although we provided the oxygen when the SpO_2_ was below 90% for at least 10 seconds in room air, monitoring of end-tidal carbon dioxide concentration by capnography could respond more quickly to respiratory depression.

## 5. Conclusion

Sedation using nonanesthesiologist-administrated propofol during ESD is safe and effective, even in patients with comorbidities who are defined as “high-risk” according to ASA physical status classification.

## Figures and Tables

**Table 1:** Baseline characteristics.

	ASA 1	ASA 2	ASA 3	*P* value
Patients (*n*)	182	273	67	
Median age (years (range))	63.5 (34.0-89.0)	72.0 (51.0-93.0)	72.0 (49.0-95.0)	<0.001
Sex				
Male/female	127/55	194/79	55/12	0.139
BMI	22.5 ± 3.09	24.0 ± 3.77	22.4 ± 3.14	<0.001
Comorbidity				
Hypertension	0	207	57	
Diabetes mellitus	0	70	23	
Heart disease	0	52	46	
Respiratory dysfunction	0	23	17	
Renal dysfunction	0	20	16	
Cerebral dysfunction	0	39	15	
Anticoagulant/antiplatelet agents (*n* (%))	9 (4.94)	75 (27.4)	39 (58.2)	<0.001
Warfarin	0	10	13	
Aspirin	3	19	17	
Clopidogrel	0	11	9	
Ethyl icosapentate	2	4	1	
Cilostazol	0	3	1	
Limaprost alfadex	3	6	0	
Heparin bridge (*n* (%))	0 (0)	21 (7.69)	16 (23.8)	<0.001
Location				0.141
Upper	44	75	23	
Middle	72	98	36	
Lower	77	125	14	
Macroscopic type				
0-I	7	7	4	
0-IIa	87	124	24	
0-IIb	14	23	2	
0-IIc	80	132	38	
0-IIa + IIc	5	11	1	
0-IIc + IIa	0	1	4	

**Table 2:** Details of propofol administration.

	ASA 1	ASA 2	ASA 3	*P* value
Mean procedure time (min)	122 ± 68.9	114 ± 66.8	109 ± 45.1	0.216
Mean induction dose of bolus injection (10 mg/10 s)	36.9 ± 14.4	36.5 ± 13.2	33.1 ± 9.9	0.166
Mean maintenance dose (mg)	176 ± 31.6	181 ± 39.1	173 ± 35.3	0.080
Mean total infusion dose (mg)	837 ± 418	791 ± 443	728 ± 266	0.218
Mean waking time (min)	14.7 ± 7.4	14.4 ± 9.8	13.8 ± 6.8	0.281

**Table 3:** Adverse events related to propofol.

	ASA 1	ASA 2	ASA 3	Total	*P* value
Propofol-related complications (*n* (%))					
At induction of bolus injection					
Hypotension	9 (4.94)	22 (8.06)	8 (11.9)	39 (7.47)	0.154
Desaturation	78 (42.8)	139 (50.9)	36 (53.7)	253 (48.4)	0.158
Bradycardia	3 (1.64)	8 (2.93)	4 (5.97)	15 (2.87)	0.194
During maintenance infusion					
Hypotension	17 (9.34)	41 (15.0)	13 (19.4)	71 (13.6)	0.075
Desaturation	5 (2.74)	4 (1.47)	2 (2.98)	11 (2.11)	0.561
Bradycardia	5 (2.74)	11 (4.02)	3 (4.47)	19 (3.63)	0.717
Resedated condition					
Hypotension	0 (0)	2 (0.73)	1 (1.49)	3 (0.57)	0.340
Desaturation	0 (0)	1 (0.37)	0 (0)	1 (0.19)	0.634
Bradycardia	1 (0.55)	3 (1.09)	1 (1.49)	5 (0.95)	0.749

**Table 4:** Adverse events related to ESD and clinicopathological tumor characteristics.

	ASA 1	ASA 2	ASA 3	Total	*P* value
ESD-related complications (*n* (%))					
Bleeding	8 (4.39)	25 (9.16)	8 (11.9)	41 (7.85)	0.075
Perforation	0 (0)	2 (0.73)	0 (0)	2 (0.38)	0.401
Aspiration pneumonia	1 (0.55)	5 (1.83)	0 (0)	6 (1.14)	0.291
Clinicopathological tumor characteristics					
Median tumor diameter (mm (range))	15.0 (3-123)	15.0 (2-81)	18.0 (5-65)		0.678
Median resected specimen diameter (mm (range))	38.0 (18-130)	38.0 (15-117)	37.0 (25-118)		0.967
Histopathological diagnosis (*n*)					
Adenoma	26	46	9		
Well differentiated	143	195	58		
Moderately differentiated	21	44	6		
Papillary adenocarcinoma	1	2	0		
Poorly differentiated adenocarcinoma	2	11	3		
m-cancer	171	252	66		
sm1-cancer	11	30	8		
sm2-cancer	6	16	4		
En bloc resection (%)	99.4	99.3	100		0.775
Complete resection (%)	96.2	96.3	94.0		0.145

## Data Availability

The database used for statistical analysis that provided data used to support the findings of this study are restricted by the Hospital Ethical Board in order to protect patient privacy. Data are available for researchers who meet the criteria for access to confidential data. More information is available from Keiichi Tominaga, MD, tominaga@dokkyomed.ac.jp.
